# Injuries and infection caused by capybara bites in a human

**DOI:** 10.1590/0037-8682-0043-2021

**Published:** 2021-03-22

**Authors:** André Luiz Rossetto, Lucas Franklin Amarante, Ana Letícia Rossetto, Vidal Haddad

**Affiliations:** 1 Universidade do Vale do Itajaí, Itajaí, SC, Brasil.; 2 Pontifícia Universidade Católica do Paraná, Curitiba, PR, Brasil.; 3 Universidade Estadual Paulista, Faculdade de Medicina de Botucatu, Botucatu, SP, Brasil.

**FIGURE 1:**
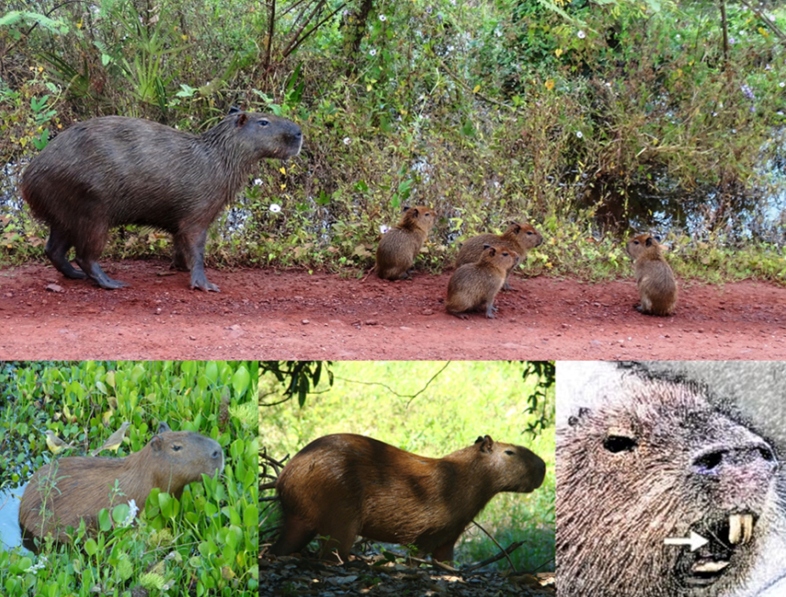
Capybaras in their natural habitat. These animals can settle in urban and peri-urban areas, increas the risk of contact with human beings. In detail, on the right: image of the incisors.

Capybaras (*Hydrochoerus hydrochaeris* - Linnaeus, 1766) are large rodents, living in groups^1^. When cornered or attacked, they defend themselves using their sharp teeth (Figure 1) ^2^. 

**FIGURE 2:**
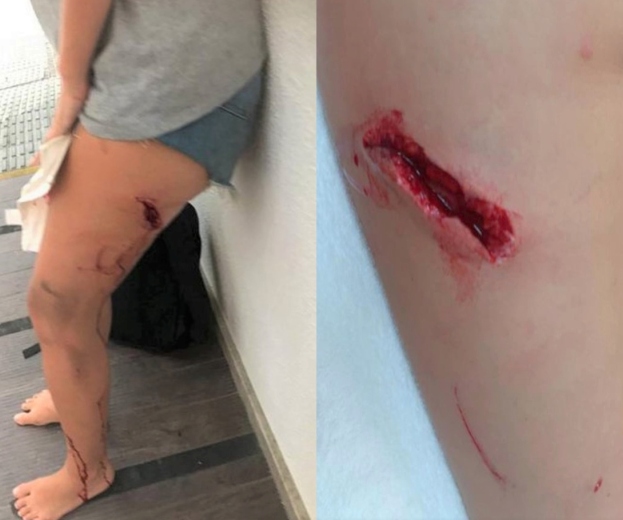
Deep laceration on the left thigh of the victim, possibily caused by the incisor teeth of a capybara, and several scratches on left leg are visible.

A 25-year-old female from Balneário Camboriú (Santa Catarina State, Brazil) was bitten on the left thigh and scratched on the left lower leg while trying to rescue her dog from a capybara attack during a walk in a forested area (Figure 2). She was successfully rescued, but the dog died two days later.

**FIGURE 3:**
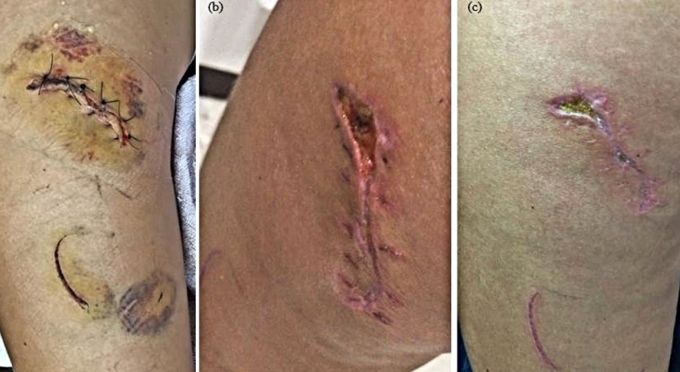
Areas of trauma due to the bite and scratches immediately after initial care (left) after 14 days, showing dehiscence and secondary infection (center), and after 25 days with a partially healed lesion (right)

The victim underwent intensive wound cleaning and suturing; she received analgesia, amoxicillin and clavulanate 2g/day for 10 days, and tetanus and rabies vaccinations. She developed an abscess in the left thigh, which was drained. After 25 days, she had scars ranging between 1 and 8 cm. At the proximal part of the left thigh, there was an approximately2.5 cm ulcer in the process of resolution and a 4.0 cm scar in the distal part of the thigh (Figure 3). Wounds and infections caused by wild animals are becoming common today which highlights the need for microbiological studies of oral flora in wild animals and traumatic structures^3^.
